# Prevalence of Color Blindness in Iranian Students: A Meta-analysis

**DOI:** 10.18502/jovr.v17i3.11580

**Published:** 2022-08-15

**Authors:** Leila Rezaei, Ehsan Hawasi, Nader Salari, Masoud Mohammadi

**Affiliations:** ^1^Department of Ophthalmology, Faculty of Medicine, Kermanshah University of Medical Sciences, Kermanshah, Iran; ^2^Department of Biostatistics, School of Health, Kermanshah University of Medical Sciences, Kermanshah, Iran; ^3^Cellular and Molecular Research Center, Gerash University of Medical Sciences, Iran

**Keywords:** Color Blindness, Color Vision Deficiency, Iran, Meta-analysis, Prevalence, Students

## Abstract

Color blindness (color vision deficiency) is a disorder that impairs the true perception of colors. Using the information in this study, appropriate policy can be made to identify high-risk groups, as well as educational policies for families to perform more effective genetic diagnosis methods.

This study aims to examine the prevalence of color blindness in Iranian students through a meta-analysis. Articles related to color blindness published between January 1990 and December 2020 were searched in Scopus, Cochrane Library, Web of Science (WoS), Science Direct, Embase, SID, MagIran, IranDoc, Medline, and Google Scholar databases. The keywords used were based on medical subject topics (MeSH Terms) and, after careful review, articles were selected according to varied sections of Participants, Exposure, Comparison, and Outcomes (PECO). Participants: students; Exposure: students with color blindness were examined; Comparison: Students from multiple provinces and regions of Iran were surveyed for color blindness; Outcomes: the pooled prevalence of color blindness in Iranian students reported from different provinces. The prevalence of color blindness in Iranian students was 3.8% (95% CI: 2.7–5.4%). The pooled prevalence of color blindness in Iranian male and female students was 4.7% (95% CI: 3.5–6.4%) and 0.7% (95% CI: 0.3–1.3%), respectively. The pooled prevalence of red–green color blindness (Tritan) was 41.7% (95% CI: 18.9–68.8%). The pooled prevalence of red color blindness (Protan) was 13.9% (95% CI: 7.8–23.8%), and the pooled prevalence of green color blindness (Deutan) based on meta-analysis was 45.3% (95% CI: 29–62.7%). Due to the high prevalence of color blindness in students, especially male students, it is necessary to be screened for through genetic tests in couples before having children.

##  INTRODUCTION

Visual impairment is one of the most common disorders affecting the students' academic performance that can directly affect their academic future.^[1]^ Some studies have addressed the psychological and social issues resulting from visual impairment problems in students.^[2,3]^

Light receptors in the human retina called photoreceptors transmit messages to the brain and enable a person to perceive various colors.^[10,11,12]^ Color perception is a response to the physical motion of a narrow handle of an electromagnetic spectrum with a wavelength of 400–700 nm, absorbed by the photoreceptors of visual pigments.^[13,14]^ This visible spectrum is only a part of the electromagnetic waves, which includes a wide range of waves with different wavelengths.^[12][13,14][15]^

Color blindness or impaired color vision is the inability to perceive the differences between some colors.^[12][13,14][15]^ It occurs when light-sensitive cells either do not perceive color signals or do not transmit them to the brain.^[14,15]^ In other words, color blindness is a condition in which the ability to distinguish some colors is considered to be less than normal.^[15]^

Many patients are unaware of the existence of their color blindness, and only during their studies or employment, based on their needs and tests, they discover that they have the color this disorder.^[13,14][15]^

The color blindness disorder is divided into congenital and acquired categories.^[4]^ Congenital color blindness is often red–green and gender-dependent, with a prevalence of 8% in men and about 0.4% in women and between 4% and 6.5% in men of Chinese and Japanese ethnicities.^[5]^ The results of this study reported that the male-to-female prevalence ratio is markedly different.^[5]^

Monochrome or complete color blindness is a rare type in which none of the cone cells are able to receive color. As a result, the person does not see any color,^[4,5]^ but if one of the three cone cells is damaged, the person does not see one of the colors. In fact, in one of the colors, the color becomes blind and the nose becomes dichromatic.^[5]^ Trichromat is one of the most common models of color blindness in which all three types of cone cells are active, but their irritability has changed.^[5]^

Dichromates usually have one of the defects of Deuteranomaly, Protanomaly, or Tritanomaly.^[6,7,8,9]^

Deuteranomaly: In this disorder, due to the proximity of the green and red spectra, the person does not see the green color correctly.^[9]^

Protanomaly: In this disorder, the cells do not process red color properly, because the wavelengths of red and green are close, and so these people see red as close to green.^[6,7,8,9]^

Tritanomaly: In this disorder, a person's sensitivity to the color blue decreases.^[6,7,8,9]^

Studies conducted in Iran on the prevalence of color blindness in students have reported different prevalence from various parts of the country. In a study conducted on students located in the city of Sari, the pooled prevalence was 2.41%, which was reported as 2.4% in male students and 0.06% in female students.^[16]^ However, a study on students from the city of Qazvin reported a pooled prevalence of 6.5%, which was 5.1% in male students and 1.4% in female students.^[17]^ In another study conducted on students from the city of Ahvaz, the pooled prevalence was 3.7%, which was 5.4% in male students and 2.1% in female students.^[18]^ Our study aims to determine the prevalence of color blindness in students through a meta-analysis.

##  METHODS

According to the Preferred Reporting Items for Systematic Reviews and Meta-Analyses (PRISMA) criteria, systematic search of databases was performed based on organizing documents for review, selection of studies, extraction and analysis.

### Search Strategy

A systematic search for articles was performed in three Iranian databases, IranDoc, MagIran, and SID using Iranian keywords, and in the international databases of PubMed, Cochrane Library, Science Direct, Scopus, Web of Science (WoS), and Embase using English keywords. The Google Scholar search engine using English keywords was also examined. The keywords used were based on medical subject topics (MeSH Terms) and, after careful review, were selected according to the varied sections of PECO.

PECO: Participants: students; Exposure: students with color blindness were examined; Comparison: students from multiple provinces and regions of Iran were surveyed for color blindness; Outcomes: the pooled prevalence of color blindness in Iranian students reported from various provinces. The search process: “color blindness", “visual disturbances", “students", “visual impairment", “color vision impairment", “visual impairment", and their Persian equivalent words and possible combinations. (AND) and (OR) operators were used for more comprehensive access to all articles. The search was conducted between January 1990 and December 2020.

(AND) and (OR) operators: (Color blindness OR Color Vision Defects OR Tritan Defect OR Retinal Diseases OR Deutan Defect OR Achromatopsia OR Protan Defect), (Visual Disturbances OR Vision Disability OR Visual Impairment), (Color Vision OR Color Perception). The word AND between keywords (Color blindness AND color vision AND students AND Iran) was used in the MeSH browser.

### Processes for Checking the Entry and Exit of Articles

Cross-sectional studies that reported the prevalence of color blindness in students were eligible for inclusion. Other observational studies as well as interventional studies and clinical trials were excluded from the study.

### Selection of Finalized Studies

The research in the mentioned databases was done independently and blindly by two researchers (EH and LR) and then the obtained articles were entered into Endnote software for secondary review as well as removal of duplicate and unrelated articles. The two researchers reviewed the articles considering the inclusion criteria; if they could not reach an agreement and decide on an article, a third researcher (MM) reviewed the article and provided the final opinion on the study.

### Quality Assessment

The quality of the studies confirmed by the STROBE checklist was assessed.^[15]^ This checklist uses 32 items to examine different aspects of the articles and rates the studies in the range of 0–32. In this review, scores > 23 to 32 were classified as high quality and grades between 22 and 14 as moderate quality.

### Data Extraction

After the search was completed, all articles with keywords such as “prevalence", “color blindness", and “Iranian student” in their titles were included in the initial list. Then a checklist including the author's name, title, year and month of the publication, place of study, age, sample size, and the pooled prevalence by sex was made. All stages of data extraction were performed by two reviewers independently.

### Statistical Review

CMA software (version 2) and Begg and Mazumdar tests and funnel plots were used to analyze the information extracted in the studies to investigate the publication bias. To evaluate and detect the degree of heterogeneity in the studies, the I^2^ heterogeneity test was used, and a meta-regression test based on population size and study time was used to investigate the effect of the heterogeneity factors.

##  RESULTS

### Literature Search

**Figure 1:**
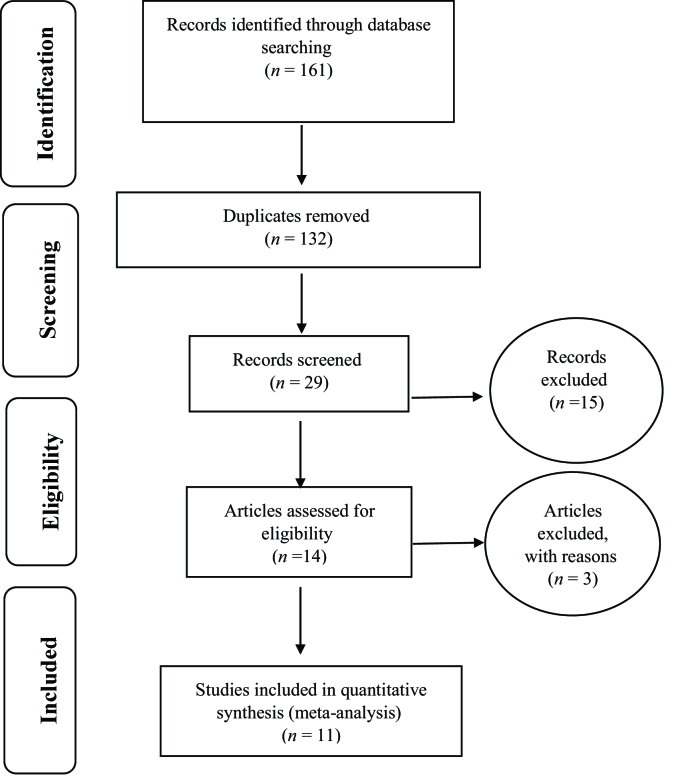
Flow chart of the review process and selection and separation of studies based on PRISMA.

Based on the PRISMA flowchart, the process of reviewing, extracting, and finalizing articles for entering into the meta-analysis was reported; consequently, 11 studies were entered into the meta-analysis. Fifteen studies were excluded from the study due to lack of relevance, and three studies due to the low quality [Figure 1; Table 1].

**Table 1:** Specifications of studies entered into the meta-analysis of the prevalence of color blindness in Iranian students


**Quality assessment**	**Prevalence (Female)**	**Sample size (Female)**	**Prevalence (Male)**	**Sample size (Male)**	**Pooled prevalence**	**Sample size**	**Participants' age**	**Area**	**Publication year**	**Author**	**Row**
Moderate	0.06	1450	2.4	1650	2.41	3000	–	Sari	2001	Farokhfar et al^[16]^	1
High	1.4	900	5.1	900	6.5	1800	9.3 ± 1.5	Qazvin	2016	Mehrpour et al^[17]^	2
Moderate	2.1	1250	5.4	1250	3.7	2500	13–18	Ahwaz	2006	Khataminia et al^[18]^	3
High	0.4	1000	3.6	1000	2	2000	–	Oromieh	2011	Sharifi et al^[19]^	4
Moderate	6.2	1166	9.5	1760	8.2	2926	11-14	Yasouj	2000	Nabavizadeh et al^[20]^	5
Moderate	–	–	–	–	5.2	500	–	Tehran	2006	Rezae et al^[21]^	6
High	0.4	1992	5.1	2408	3	4400	–	Mashhad	2011	Ostadi et al^[22]^	7
Moderate	0.2	1414	3.3	1414	1.7	2828	–	Kermanshah	2000	Omidian et al^[23]^	8
Moderate	0.35	–	7	–	4.4	706	–	Hamadan	1996	Ramezani et al^[24]^	9
Moderate	–	–	–	–	8.7	3000	–	Zanjan	2006	Hossaini et al^[25]^	10
Moderate	0.33	1500	4.2	1500	2.3	3000	–	Isfahan	2001	Azarian et al^[26]^	11
	
	

### Investigation Bias in Studies

The result of the I^2^ test was 97.3%, and a random-effects model was used to meta-analyze the results of the studies. The results of publication bias were measured using the Begg test and were not statistically significant (*P* = 0.212).

### The Pooled Prevalence of Color Blindness

**Figure 2:**
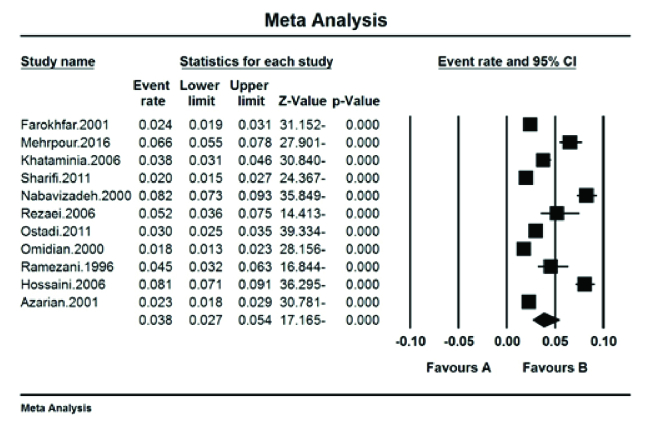
The prevalence of color blindness in Iranian students.

In 26,660 people in the age range of 7–18 years, the pooled prevalence of color blindness in Iranian students was 3.8% (95% CI: 2.7–5.4%) [Figure 2].

### The Prevalence in Male Students

**Figure 3:**
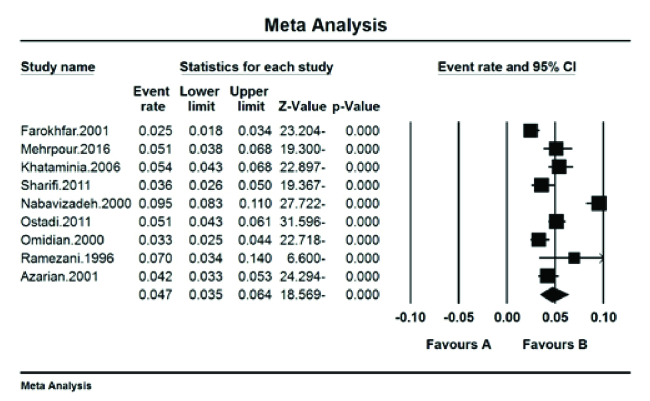
The prevalence of color blindness in Iranian male students.

The total number of samples included in the study was 11,982 people. Based on the meta-analysis, the pooled prevalence of color blindness in Iranian male students was 4.7% (95% CI: 3.5–6.4%) with the I^2^ test (92.4%). The highest prevalence of color blindness was reported in the city of Yasuj with 9.5% (95% CI: 8.3–11%) in 2000,^[20]^ and the lowest prevalence of color blindness was reported in the city of Sari with 2.4% (95% CI: 1.8–3.4%) in 2001^[16]^ [Figure 3].

### The Prevalence in Female Students

**Figure 4:**
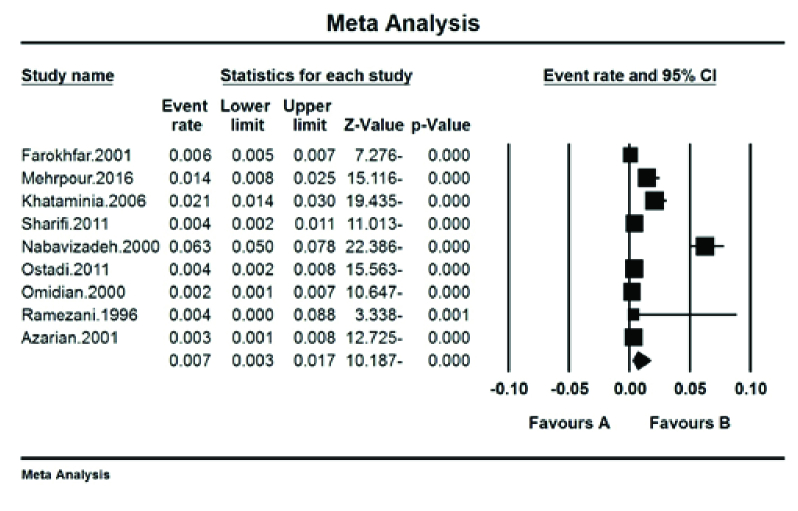
The prevalence of color blindness in Iranian female students.

The pooled prevalence of color blindness reported was 0.7% (95% CI: 0.3–1.7%) with I^2^ test (95.04%). The highest color blindness was reported in the city of Yasuj with 6.2% (95% CI: 5–7.8%) in 2000,^[20]^ and the lowest rate of color blindness was in the city of Sari with 0.06% (95% CI: 0.05–0.07%) in 2001^[16]^ [Figure 4].

### Color Blindness by Its Types

**Figure 5:**
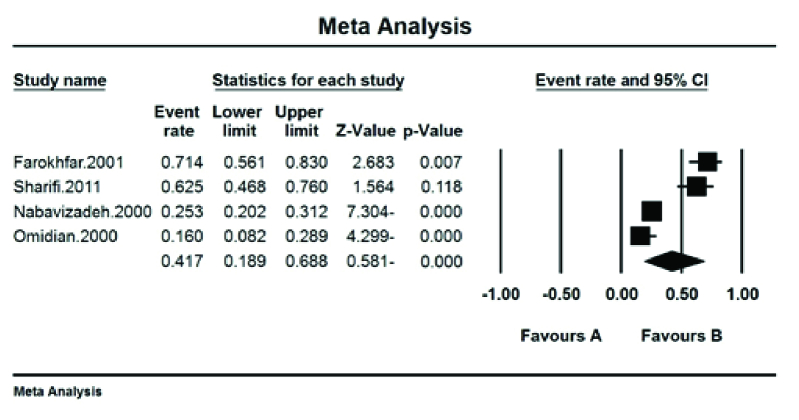
The prevalence of red–green color blindness (Tritan) in Iranian students.

**Figure 6:**
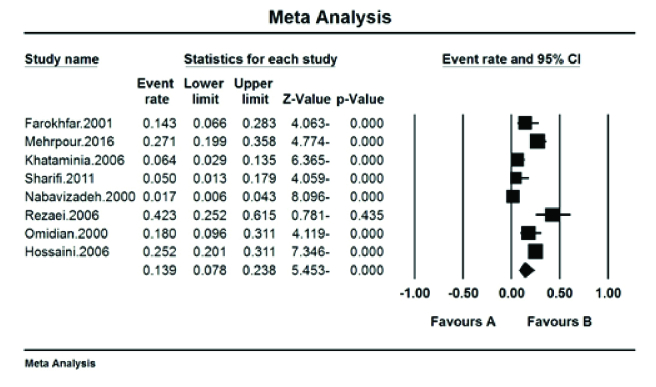
The prevalence of red color blindness (Protan) in Iranian students.

**Figure 7:**
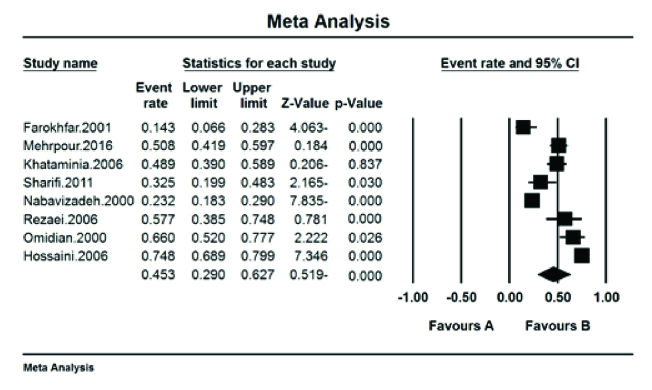
The prevalence of green color blindness (Dutan) in Iranian students.

The prevalence of red–green color blindness (Tritan) in Iranian students was 41.7% (95% CI: 18.9–68.8%) with the I^2^ test (93.8%) [Figure 5]. The prevalence of red color blindness (Protan) in Iranian students was 13.9% (95% CI: 7.8–23.8%) with the I^2^ test (88.01%) [Figure 6]. The prevalence of green color blindness (Dutan) in Iranian students was 45.3% (95% CI: 29–62.7%) with the I^2^ test (95.01%) [Figure 7].

**Figure 8:**
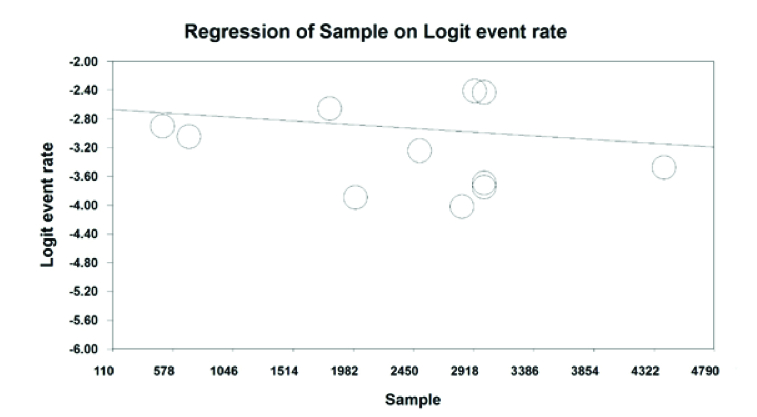
Heterogeneity check diagram of the color blindness by sample size.

**Figure 9:**
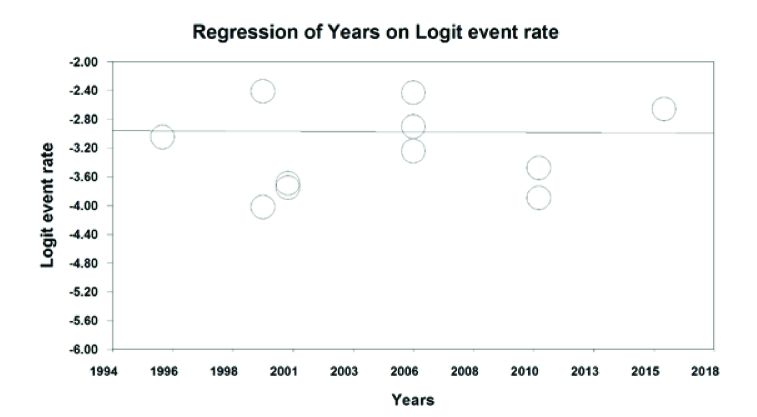
Heterogeneity check diagram of the color blindness by the year of research.

According to Figure 8, color blindness in students decreases with an increasing sample size (*P* < 0.05). Furthermore, according to Figure 9, overall color blindness in students increases with the length of the research year but is not statistically significant (*P* = 0.765).

**Figure 10:**
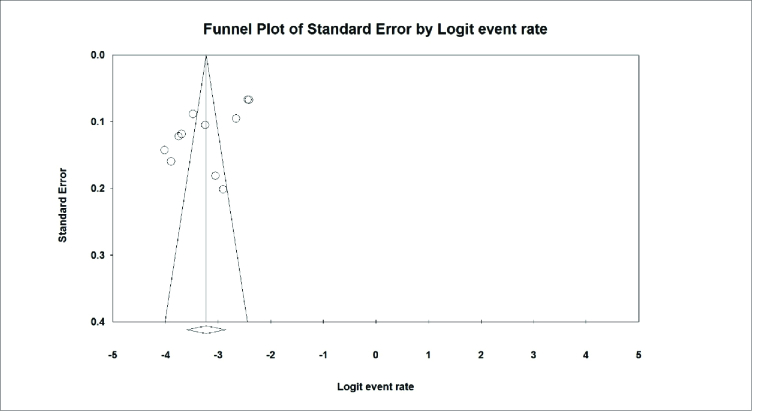
(Funnel plot) The results of the prevalence of color blindness in Iranian students.

The Begg test measured the results of the publication bias in the studies and the Mazumdar rank correlation test, according to the large sample size entered in the studies. The publication bias was not statistically significant (*P* = 0.275) [Figure 10].

##  DISCUSSION

Understanding the epidemiology of a disease can help in addressing it on a macro level.^[27]^ Although color blindness is not a very common or dangerous disease, such a complication in students can have psychological effects on their educational status and future careers. Therefore, this disorder presents a significant challenge when addressing the effects of medical disorders on the educational success in students.

Based on our analysis, the prevalence of color blindness in Iranian students was reported as 3.8%, and the color blindness in Iranian male and female students was reported as 4.7% and 0.7%, respectively. Studies show that color blindness affects 1 in every 12 men and 1 in every 200 women.^[28]^ One in six people carries the color blindness gene, which is passed on to their son causing color blindness.^[28]^ Due to the genetic nature of this disease, it was revealed that the greater the number of consanguineous marriages in the population, the higher the prevalence of color blindness.^[28]^

The results of the prevalence of the current study was higher than the color blindness reported as 2.1% in Nepal,^[29]^ 2.6% in Nigeria,^[30]^ and 1% in Bangladesh.^[31]^ In studies conducted in Europe and Spain, the prevalence was 4.02% in boys and 0.46% in girls.^[33][34][6,7,8,9]^

In a study by Khataminia et al, it was reported that approximately 6% of men of European descent have color vision problems, compared with 4.9% of men and 0.64% of women in Asian countries.^[18]^ This ratio is 3.1% in men and 0.7% in women of African countries, Native American or Mexican descent.^[18,28]^

In a study reported in Africa and Ethiopia, the prevalence was 4.2% in boys and 0.2% in girls.^[35]^ In a United States study, a prevalence of 2.6% was reported in boys.^[36]^ A study in Australia reported that 8% of men and 0.4% of women have color blindness.^[17]^

In the present study and in reviewing the meta-regression results, it was reported that with an increasing sample size, the prevalence of color blindness in Iranian students decreases, and the prevalence of color blindness in Iranian students increases with increasing research years.

Methods used for determining the disorder and tools needed to investigate the disorder are not the same among different studies. Multiple tests have been utilized to perform research including the use of Ao-HR. R and Pseudoisochromatic tests have been common^[16,37]^ since most studies have used the Ishihara booklet.^[16]^ However, different studies have mentioned the number of people surveyed and different age groups in justifying the significant differences in various studies.^[16]^

The pooled prevalence of red–green color blindness (Tritan) in Iranian students was 41.7%, the pooled prevalence of red color blindness (Protan) was 13.9%, and the pooled prevalence of green color blindness (Deutan) based on the meta-analysis was 45.3%. This is based on the opinion of the American Ophthalmological Society reports' red–green vision deficiency in 8% of men and 0.5% of women.^[18]^

A study conducted in Croatia reported that 8.4% of the participants were color blinded, of which 1.2% were Protan and 4.2% were Deutan. ${}^{\left[17,18\right]}$ The Deutan in Ethiopia was 3.4%,^[35]^ in Turkey 2.2%,^[38]^ in Saudi Arabia 1.9,^[39]^ and in a Spanish study 2.1%.^[40]^

Although color blindness is almost ineffective in most job activities, due to its high prevalence, especially among students, it can affect their academic performance and limit their academic achievement, thus increasing public awareness through the mass media. And social media is essential to enhance genetic testing in couples before their children are born. Parents of children should also be informed about color vision disorders in their child's eyes so that with early diagnosis, specific treatment measures can be performed. Also, since there is no effective treatment for color blindness, the psychological damage of this disorder can be reduced by reassuring individuals and students that color blindness does not affect their future careers, as well as providing their own education in schools. Based on the information obtained, it is recommended that periodic screening of students for early detection of color blindness along with parental training to identify children who have color blindness symptoms should be performed.

### Strengths and Limitations 

The most important strength of this research is that it is the first Iranian meta-analysis and provides accurate population-based information. An important limitation of the study is the limited number of studies conducted in Iran.

##  CONCLUSION

Due to the high prevalence of color blindness in students, especially male students, it is necessary to take appropriate measures through appropriate screening for genetic tests in couples and parents who have color-blind children, screening parents with color blindness, as well as genetic counseling for parents who have a color-blind child and planning to conceive again. This study also reports that the prevalence of different types of color blindness in students is high and requires continuous follow-up and screening.

##  Ethics Approval

The study protocol was approved by the Vice Chancellor for Research and Technology of Kermanshah University of Medical Sciences with the following code: IR.KUMS.REC.1400.009.

##  Financial Support and Sponsorship

This study was funded by the Vice-Chancellor for Research and Technology of Kermanshah University of Medical Sciences. This fund had no effect on the process of conducting the present study.

##  Conflicts of interest

The authors declare that they have no conflict of interest.
